# A comprehensive survey of techniques for developing an Arabic question answering system

**DOI:** 10.7717/peerj-cs.1413

**Published:** 2023-06-08

**Authors:** Yazeed Alkhurayyif, Abdul Rahaman Wahab Sait

**Affiliations:** 1 Department of Computer Science, Al Quwayiyah College of Sciences and Humanities, Shaqra University, Saudi Arabia; 2 Department of Documents and Archive, Center of Documents and Administrative Communication, King Faisal University, Al-Ahsa, Saudi Arabia

**Keywords:** Deep learning, Arabic question-answering system, Machine learning, Arabic chatbot, Interactive question-answering system

## Abstract

The question-answering system (QAS) aims to produce a response to a query using information from a text corpus. Arabic is a complex language. However, it has more than 450 million native speakers across the globe. The Saudi Arabian government encourages organizations to automate their routine activities to provide adequate services to their stakeholders. The performance of current Arabic QASs is limited to the specific domain. An effective QAS retrieves relevant responses from structured and unstructured data based on the user query. Many QAS studies categorized QASs according to factors, including user queries, dataset characteristics, and the nature of the responses. A more comprehensive examination of QASs is required to improve the QAS development according to the present QAS requirements. The current literature presents the features and classifications of the Arabic QAS. There is a lack of studies to report the techniques of Arabic QAS development. Thus, this study suggests a systematic literature review of strategies for developing Arabic QAS. A total of 617 articles were collected, and 40 papers were included in the proposed review. The outcome reveals the importance of the dataset and the deep learning techniques used to improve the performance of the QAS. The existing systems depend on supervised learning methods that lower QAS performance. In addition, the recent development of machine learning techniques encourages researchers to develop unsupervised QAS.

## Introduction

Across the globe, users face challenges in extracting meaningful responses from the question-answering system (QAS) (Loginova, Varanasi & Neumann, 2021; Alwaneen et al., 2022; Alamir et al., 2021). The user query and context are crucial factors in evaluating the QAS performance (Bessaies, Mesfar & Ben Ghzela, 2018). Recent developments in internet technology have increased the volume of unstructured data (Fuad & Al-Yahya, 2022). The purpose of an Information Retrieval System (IRS) is to find relevant materials that answer a user’s query (Hamza et al., 2021). However, users face challenges in identifying a straightforward solution for their queries. Therefore, the reliability of the data became crucial. Search engines provide results based on the user query (Arbaaeen & Shah, 2021). There is a demand for developing QAS using natural language processing (NLP) techniques. An NLP-based QAS offers a concise response to a user query. In addition, open-domain QASs can be used as a search engine for retrieving responses from a large text corpus (Malhas & Elsayed, 2020).

QAS presents a concise response from the massive text corpus using the natural language-based user query. A bag of words model is employed in QAS to retrieve the relevant resources (Ben-Sghaier, Bakari & Neji, 2019; Romeo et al., 2019; Zeid, Belal & El-Sonbaty, 2020). Due to the vast quantity of data, QAS returns false positive results and users may not be satisfied with the outcome. Due to this limitation, manual interaction is used to acquire information, but it takes more computation time  (Faris et al., 2022). An ideal QAS response is in natural languages that satisfy the user’s response (Mozannar et al., 2019). The ultimate goal is to provide an appropriate response promptly and effectively.

NLP and information retrieval (IR) are rapidly developing in machine learning (ML) research (Lahbari, El Alaoui & Zidani, 2018). As a method of data analysis, machine learning enables the development of automated analytical models. ML-based systems can analyze large amounts of data, find meaningful patterns, and act autonomously. On the other hand, NLP is a combination of linguistics and AI. It improves communication between humans and machines. In addition, it integrates mathematics and data to build systems to interpret natural language. It examines the grammatical structure of phrases and specific meanings of words and utilizes algorithms to extract meaning and deliver outputs. In other words, it understands human language to carry out various activities without human intervention. Learning and comprehending a natural language is difficult. The three main parts of QAS are questions, documents, and answer extraction (Longpre, Lu & Daiber, 2021). An approach (Abuleil & Evens, 2004) was introduced for extracting names from Arabic texts. A database and graphs were developed to represent the words that might reflect the names. Initially, the phrases were marked, and further, the relationships between the phrases were identified.

NLP-based QAS comprises three critical stages to respond to a user query (Al-Shenak, Nahar & Halawani, 2019). In the first stage, the question is analyzed in terms of its syntax and semantics to extract the user’s intent, highlight the keywords, and generate the inquiry. This part of the question exposes its focus or primary purpose (Samy, Hassanein & Shaalan, 2019). Furthermore, it determines the category of questions based on a predetermined classification and the expected response type. NLP has many techniques, including named entity recognition (NER) and classification algorithms (Breja & Jain, 2022). In the second stage, documents are navigated. The purpose of a search engine is to find the most relevant content from a large pool of results. It employs a complex processing technique to sort valuable answers (Alothman & Wahab Sait, 2022). The final step is locating content that may provide valuable insights. In addition, recent quality assurance systems are used to justify and refined the retrieved responses.

Recent studies reveal that 450 million Arabs comprise around 10% of the 1.8 billion Muslims using Arabic (Hao et al., 2022). Therefore, there is an exponential growth of Arabic-speaking Internet users. For instance, during the past 20 years, the number of Arabic-speaking Internet users has increased by 9348%, whereas the number of English-speaking users has increased by only 742.91% (Gemirter & Goularas, 2021).

In recent literature (Almiman, Osman & Torki, 2020; Utomo, Suryana & Azmi, 2020; Mutabazi et al., 2021; Boudjellal et al., 2021), researchers have reported the types of QASs and their features. However, the classification of QASs and the challenges of their implementation have not been satisfactorily addressed. Most studies have focused on the general architecture of QASs, and only a few studies have been concerned with the recent deep learning (DL) techniques related to QAS development. In addition, information on the challenges and limitations of QAS development is scarce.

There is a need for a systematic literature review on QAS regarding techniques. Therefore, this research investigates recent techniques for developing Arabic QASs. The proposed study categorizes QASs into multiple categories to support researchers and developers.

This study is expected to provide:

1. A detailed account of the recent Arabic QAS.
2. A classification of QASs based on their underlying methods.
3. Benchmark evaluation techniques for assessing the performance of QAS.

The rest of the study is organized as follows: section 2 highlights the background of the Arabic QAS. The methodology of the proposed review is described in section 3. Sections 4 and 5 present the outcome of the review. Finally, section 6 concludes the study with its future direction.

## Background

There are several varieties of the Arabic language, including classical Arabic, modern standard Arabic (MSA), and regional dialects (Loginova, Varanasi & Neumann, 2021; Alwaneen et al., 2022; Alamir et al., 2021). Arabic is a phonetic language with 28 fundamental letters. Every letter has the potential to take four distinct forms, and these variations are determined by the letter that came before it (Bessaies, Mesfar & Ben Ghzela, 2018; Fuad & Al-Yahya, 2022; Hamza et al., 2021; Arbaaeen & Shah, 2021). Furthermore, Arabic has an extensive vocabulary. These complexities require special processing beyond the capabilities of standard NLP systems. Arabic NLP research is challenging due to lacking linguistic resources, including corpora, dictionaries, and lexicons (Malhas & Elsayed, 2020). Arabic QAS development requires corpora that contain a variety of queries as well as documents to train the systems (Ben-Sghaier, Bakari & Neji, 2019). Many researchers use the customized dataset to overcome the difficulty in locating existing datasets. There are limited Arabic datasets to train and test the Arabic QAS (Romeo et al., 2019).

The Arabic language has several derivative forms. Forming the root verb takes three or four letters (Zeid, Belal & El-Sonbaty, 2020). All adjectives are derived from verbs and they are also inferences. Given the prevalence of logical templating in Arabic deductions, the relationship is as follows: Lemma = Root + Pattern (Faris et al., 2022). Furthermore, in the case of a general conclusion, individuals should understand the meaning of Lemma. The initial stage of Arabic text analysis involves producing tokens or individual words from the input phrase (Mozannar et al., 2019). A segmentation error may occur if the tokenization process incorrectly identifies a component of a term as a prefix or suffix rather than a part of the Lemma (Lahbari, El Alaoui & Zidani, 2018). The issue emerges in NER when the n-grams at the end of a word are divided into different tokens because they were incorrectly interpreted as objects or personal/possessive anaphora (Al-Shenak, Nahar & Halawani, 2019).

Furthermore, some inaccurate tags could be generated using word embedding tools. A question classification method (Al Chalabi, Ray & Shaalan, 2015) was developed for Arabic QAS. In this experiment, regular expressions and context-free grammar were utilized. To design the logical expressions, NOOJ was used.

NER is the first step toward answering factual inquiries (Samy, Hassanein & Shaalan, 2019). During this step, the recognizer is responsible for extracting the names of persons and places. Capitalization is not used in Arabic, which increases the difficulty of performing NER tasks. Lack of capitalization thus adds a lot of ambiguity for parsing queries and answer formation (Breja & Jain, 2022; Alothman & Wahab Sait, 2022; Hao et al., 2022). Due to their unique characteristics, proper nouns may require specialized hardware for appropriate recognition. In another approach (Al Chalabi, 2015), question answering was divided into three phases: question analysis, document analysis, and answer analysis. To conduct the experiment, NOOJ, and Arabic wordnet were used. Similarly, the QAS had three phases (Biltawi, Tedmori & Awajan, 2021): question analysis, answer extraction, and information retrieval. The authors examined the gap in Arabic question answering utilizing six different datasets.

The Arabic language presents an additional morphological difficulty in making two-word compounds. This conjunction is flexible since it may be used with nouns, verbs, or particles (Gemirter & Goularas, 2021). Though it is rarely heard in classical Arabic, it is a part of MSA. As its name suggests, anaphora resolution generates complexity between pronouns and nouns (Longpre, Lu & Daiber, 2021). To properly understand the meaning and function of an anaphor, it is crucial to identify its predecessor. In written and verbal communication, anaphora is quite common. A QAS termed IDRAAQ was proposed (Abouenour, Bouzoubaa & Rosso, 2012). Multilevel preprocessing was adopted to enhance the quality of the retrieved passage. This approach was based on keywords and structure levels.

The complexity of Arabic morphology stems from the fact that there are about 10,000 separate roots (Almiman, Osman & Torki, 2020). The study of Arabic morphology reveals 120 distinct patterns (Utomo, Suryana & Azmi, 2020). Mutabazi et al. (2021) discuss the significant role of 5,000 different origins in Arabic morphology. In Arabic, the order of words can be switched around. Users can select the word that they feel should serve as the sentence’s focus and place it at the beginning of the sentence (Utomo, Suryana & Azmi, 2020; Mutabazi et al., 2021; Boudjellal et al., 2021). An approach for query expansion in the Arabic language was proposed (Al-Chalabi, Ray & Shaalan, 2015) based on semantics. The semantically equivalent keywords were added through semantic sources in questions. The proposed approach produced highly accurate answers. Deep learning methods were used for open-domain question answering (Alsubhi, Jamal & Alhothali, 2022) in the Arabic language. Dense passage retrieval was used to retrieve the passage, and AraELECTRA was used for reading the passage. The results revealed that the proposed approach outperformed the existing TF-IDF approach. Similarly, a student question-answering system (Abdelhafez, Khateeb & Yahya, 2022) was developed for Arabic query auto-completion. The dataset used in the research was directly collected from students. The results achieved by the system were encouraging.

In QAS, the syntactic analyzer receives the input tokens from the lexical analyzer and applies Arabic grammar rules to determine the sentence structure. Due to the considerable freedom of word order in an Arabic phrase, syntactic ambiguities arise, necessitating an examination of all conceivable grammatical rules and agreement between elements (Ahmed, Ahmed & Anto, 2017).

A language’s semantic level is concerned with the meaning of words and their relationships. Polysemy and homonymy are the most common forms of binary verbal interactions in different languages (Mohammed, Nasser & Harb, 1993). Homonyms are words with similar meanings. They are not linked to another word in any phonological or morphological manner. The symmetrical group contains synonyms and antonyms, whereas the hierarchical group contains holonyms and meronyms (Hammo et al., 2002; Brini et al., 2009b; Brini et al., 2009a).

An ontology-based question-answering approach was developed (Sheker et al., 2016) for Islamic Fatwa. For this purpose, the authors used TF-IDF. The proposed approach achieved a 94% F-measure score. Another approach by Ahmed, Babu & Anto (2017) used multiple techniques such as a parser and POS tagger for the Arabic language. Further, they employed named entity recognition, tokenization, removal of stop words, expansion of questions, and classification of questions. Four different important elements of questions were identified. The reciprocal rank method was used to compute the mean of the documents. Bdour & Gharaibeh (2013) outline the development of Arabic QAS and present the difficulties encountered by these systems. Furthermore, they categorized the Arabic QAS based on their functionalities. Trigui, Belguith & Rosso (2010) discussed the importance of closed-domain (limited) and open-domain (non-factoid) Arabic QAS. To develop collaborative Arabic QAS, the authors emphasized the significance of harnessing social media data and blogs and creating testbeds for QAS development. Zheng (2002) conducted a study in which analyzed and contrasted eleven quality assurance procedures. They compared Arabic QASs according to criteria such as domain, programming language, WordNet usage, ontology usage, linguistic resource usage, methodology, dataset source usage, answer form, question type, features, and experimental outcomes (Kurdi, Alkhaider & Alfaifi, 2014). They also classified the QAS based on its characteristics.

An ML strategy for answering questions on Arabic trivia is suggested by  Azmi & Alshenaifi (2017). A support vector machine (SVM) was used in this system to classify questions and select appropriate answers. It extracts features from the queries to identify an optimal response. The question class combines unigrams, bigrams, wh-words, and topic headwords into a single category.

**Figure 1:**
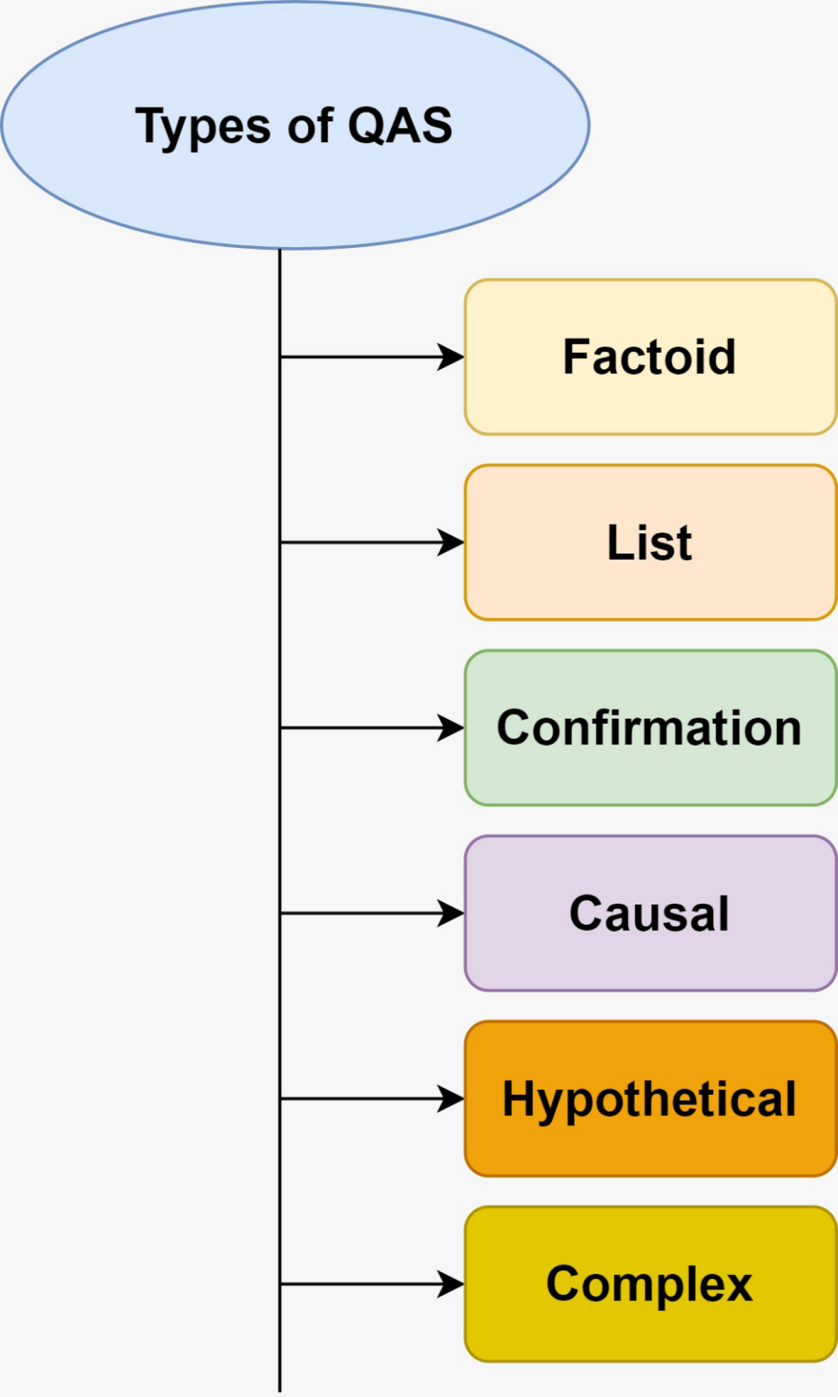
Types of QAS.

Figure 1 displays the types of QAS. There are six types of QAS: factoid, list, confirmation, causal, hypothetical, and complex. The difficulty of answering users’ queries depends on the nature of the queries. Therefore, the responses provided by the QAS are closely related to the categorization of the questions. Misclassifying questions in QASs accounts for 36.4% of mistakes (Reddy & Madhavi, 2017). Albarghothi, Khater & Shaalan (2017) organize questions based on a fine-grained content-based categorization. They categorized QAS using the functional requirements.

### Factoid QAS

The response of factoid QAS is often considered adequate. Questions of the “factoid” variety are typically part of a vast dataset  (Alothman & Wahab Sait, 2022; Brini et al., 2009b; Fareed, Mousa & Elsisi, 2013). Answering inquiries of the “factoid” variety does not require sophisticated NLP systems. Properly identifying and sub-classifying questions are critical factors of the factoid QAS. Brief statements describing entities, people, dates, and places are typical responses to queries of the “factoid” type.

### List QAS

Any replies to this query should be presented as a list (Alothman & Wahab Sait, 2022; Brini et al., 2009b; Fareed, Mousa & Elsisi, 2013). For instance, list the cities in Saudi Arabia, and the query can list the name of the cities in the country. In the case of list-style queries, the entities are selected as named entities. Consequently, the results of list-based inquiries can be informative. Answering list-style queries does not require extensive NLP-based QAS. Strategies used for “factoid” questions can be applied to “list” queries. A common feature of list-style questions is providing a minimum required amount of a particular object or number (Fareed, Mousa & Elsisi, 2013).

### Confirmation QAS

Answering confirmation queries with a yes or no requires understanding the inference process and the reasoning ability (Alothman & Wahab Sait, 2022; Brini et al., 2009b; Fareed, Mousa & Elsisi, 2013). Some experienced users require common reasoning and world knowledge for new understanding. Users can benefit from public opinion included in opinionated data sources for their perceptions of a product. An issue with opinion-based inquiries is that they can be compromised by spam or false news-detecting software, which hampers actual opinion mining  (Fareed, Mousa & Elsisi, 2013).

### Causal QAS

Unlike the responses to factoid-style inquiries, causal QAS does not refer to specific people, places, or things (Alothman & Wahab Sait, 2022; Brini et al., 2009b; Fareed, Mousa & Elsisi, 2013). It is necessary to provide descriptive solutions to queries of causation. Users seeking information about the causes of things will often raise “why” queries. The explanations of causal inquiries might range in length from a few words to many sentences.

### Hypothetical QAS

There are no hard and fast rules in responding to a hypothetical question. The phrase “what if” is frequently used to introduce hypothetical situations (Alothman & Wahab Sait, 2022; Brini et al., 2009b; Fareed, Mousa & Elsisi, 2013). This type of question has poor dependability and accuracy and is highly dependent on the users and the context.

### Complex QAS

Answers to more complicated queries typically form a bulleted list of key points (Alothman & Wahab Sait, 2022; Brini et al., 2009b; Fareed, Mousa & Elsisi, 2013). To find solutions to complex problems, elaborate methods should be employed. Answering a difficult question requires filtering diverse data. Each component of the complicated inquiry is designed to elicit different answers from various sources.

## Methodology

The authors focus on the techniques of QAS development. In addition, they intend to classify the studies based on the QAS methods. The authors (Loginova, Varanasi & Neumann, 2021; Alwaneen et al., 2022; Alamir et al., 2021; Bessaies, Mesfar & Ben Ghzela, 2018; Fuad & Al-Yahya, 2022) asserted the importance of analyzing the findings using the systematic mapping approach. The authors use the systematic mapping approach as the methodological procedure (Romeo et al., 2019). The review process proceeds from formulating the research questions, defining the search process, setting the criteria for filtering the findings to categorizing the findings.

Recent studies focus on QAS to process the users’ natural language queries. English QAS has achieved high accuracy in the last several decades. However, Arabic QAS is in its infancy (Hamza et al., 2021; Arbaaeen & Shah, 2021; Malhas & Elsayed, 2020). Conducting a literature review to evaluate the current state of Arabic QAS and offer potential solutions is essential. The general and focal research questions are in Table 1. The difference between general and focal questions is that the former are concerned with more general subject components, such as the characteristics and broader methods used for the Arabic QAS. Research studies on QAS challenges and limitations were gathered, analyzed, and discussed. The studies were collected from the years 1993 to 2022. In contrast, FQs are focused on addressing topics like approaches to ML for the QAS architecture. Table 2 presents the significant terms and search strings used for collecting the research articles.

**Figure 2:**
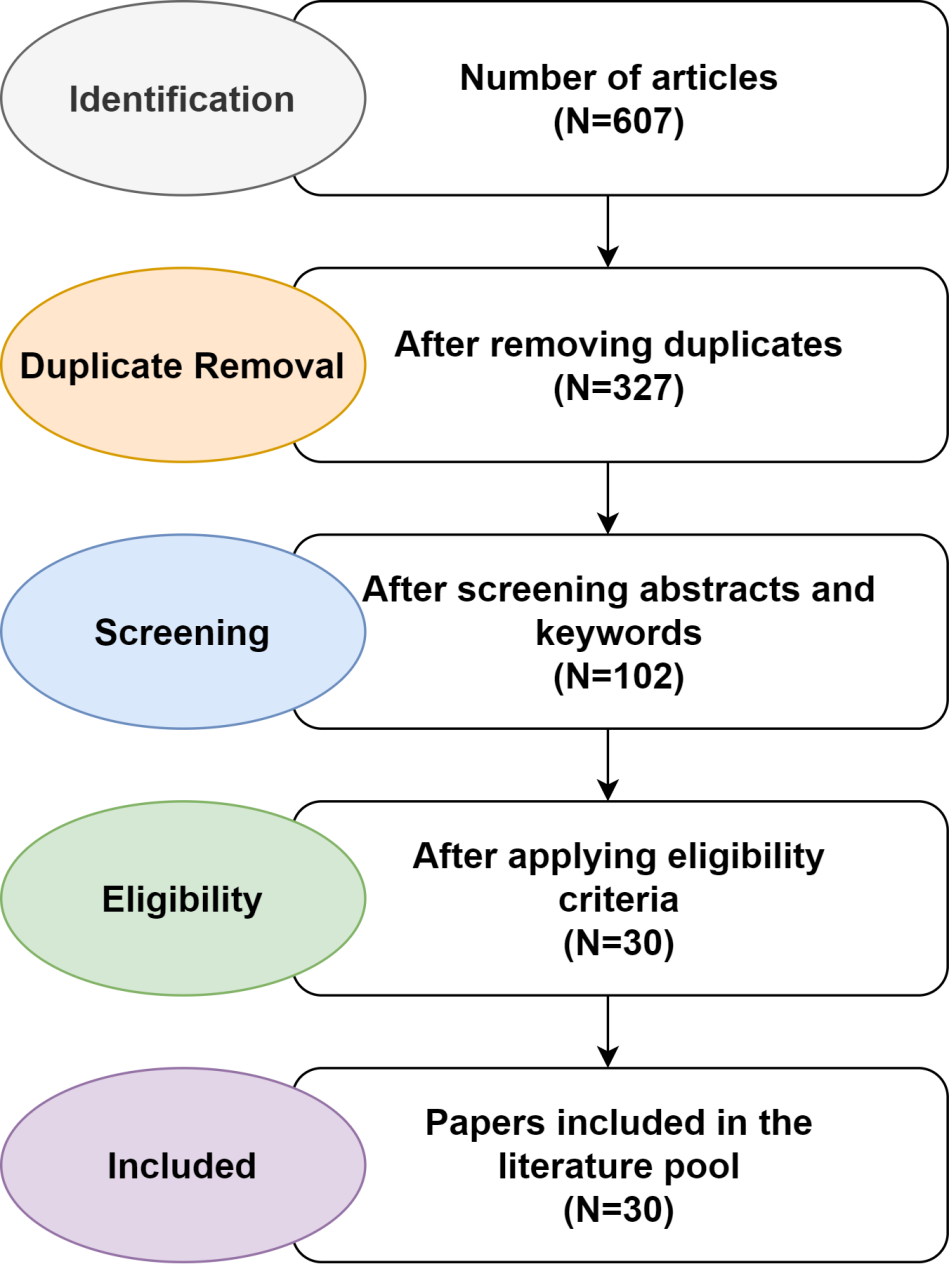
Paper selection process using PRISMA guidelines.

The majority of the publications were collected through the Google Scholar search engine using phrases such as “Arabic question answering”, “question answering systems”, “answering Arabic questions”, “Arabic question answering techniques”, “Arabic question answering methods”, and “machine learning techniques”, “Arabic chatbot”, and “Arabic question answering framework”. A sum of 607 articles was extracted and only 30 papers were chosen in line with the selection criteria presented in Table 3. Figure 2 shows how papers were selected using the PRISMA guidelines. Only publications with “Arabic” and “question answering system” in their title, abstract, or list of keywords were considered for inclusion in the review. The criteria used to select articles are listed in Table 4. The authors filtered the irrelevant studies and selected the studies most relevant to the primary research questions.

**Table 1:** Research questions.

Type	Questions
General	What are the features of Arabic QAS?
	What are the widely used techniques in QAS development?
	How does artificial intelligence influence the Arabic QAS?
Focal	Is there any development in the recent Arabic QAS?
	Is there any relationship between deep learning techniques and the Arabic QAS?
	What are the evaluation techniques used for performance evaluation?

**Table 2:** Search strings based on significant terms.

Major terms	Search strings
Arabic QA System	(Arabic question answering OR Arabic QA systems OR interactive Arabic QA systems)
Machine Learning	(Machine learning OR artificial intelligence AND Arabic Chatbot)
Deep Learning Techniques	(Deep learning techniques or automated Arabic human interaction)

**Table 3:** Inclusion and exclusion criteria.

Type of Criteria	Criteria
Inclusion	Publications in conferences and peer-reviewed journals.
	Full content relevant to the Arabic QA systems and machine learning techniques.
Exclusion	Publications are relevant to the Arabic QAS.
	Publications in non-English languages.
	Duplicate publications.
	Theses, dissertations, abstracts and books.

The selection criteria are used to evaluate the research articles that developed an Arabic QAS or a resource for the Arabic QAS. Accordingly, a total of 30 papers conducted from 1993 to 2022 were included in the review. Only eight of the 30 publications targeted Arabic QA datasets, while the other 22 presented other QA systems. Table 4 outlines the details of the studies and databases.

## Results and Discussion

Researchers encounter many limitations in developing new systems and techniques for Arabic QAS. Arabic NLP may be accomplished *via* several different resources. Word analysis tools are essential resources for deciphering the linguistic structure of words. Arabic is a highly inflectional and derivational language (Al-Shenak, Nahar & Halawani, 2019; Mohammed, Nasser & Harb, 1993; Brini et al., 2009b; Ray & Shaalan, 2016). The phrase is rarely presented in its exact form. New affixes may dilute the content and make it harder to perform tasks like question analysis and passage recollection.

**Figure 3:**
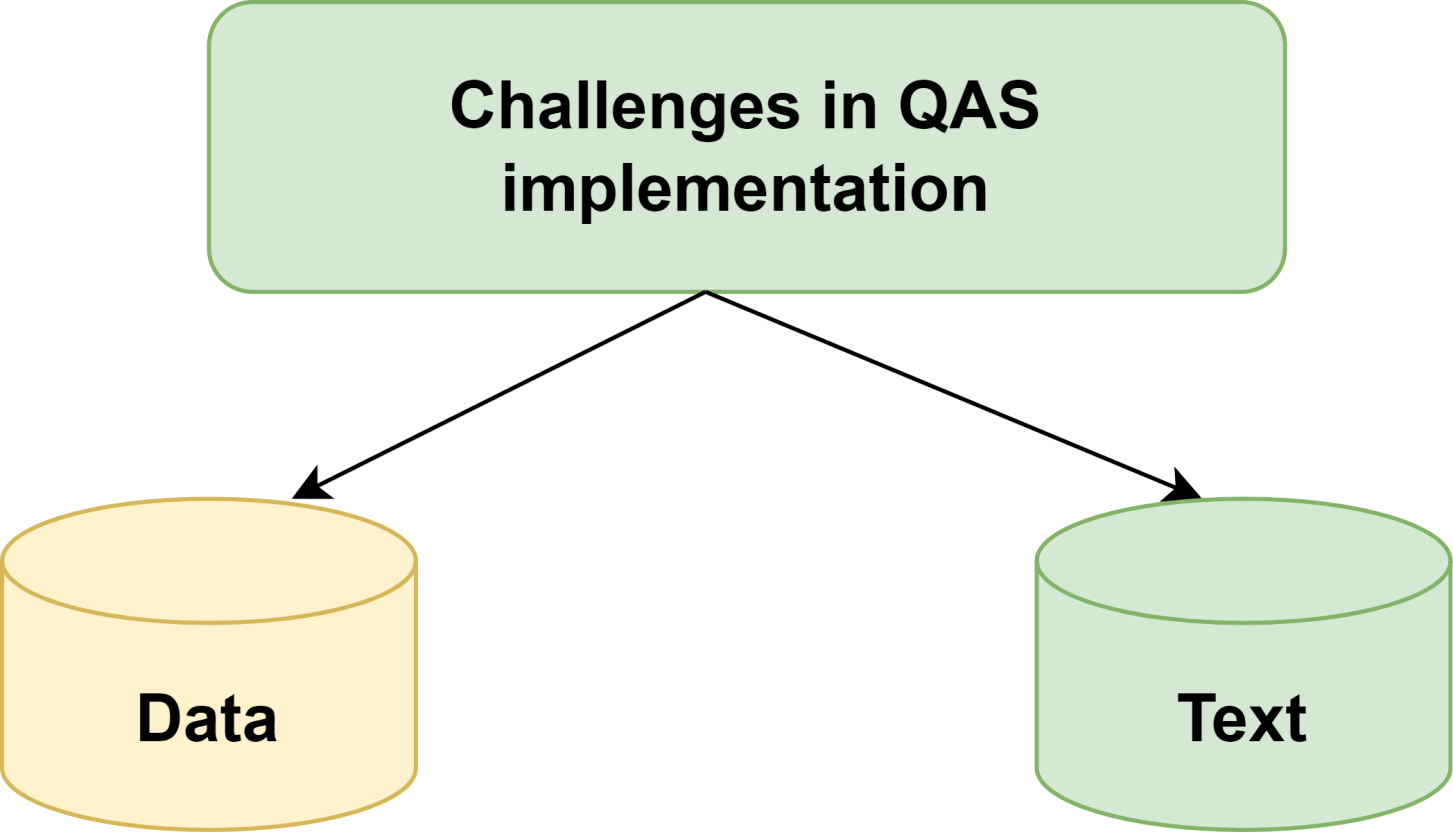
Challenges in QAS implementation.

Figure 3 represents the challenges of implementing the QAS in any organization. These challenges include text and data. Data ambiguity can occur if we interpret the data with textual description (Loginova, Varanasi & Neumann, 2021; Alwaneen et al., 2022; Alamir et al., 2021; Bessaies, Mesfar & Ben Ghzela, 2018; Fuad & Al-Yahya, 2022; Hamza et al., 2021; Arbaaeen & Shah, 2021). Identifying the relationship between entities and objects is important to extract effective information. Semantics is essential to determine the relationships between entities and objects. Unless the context and semantics of interaction are recognized, entities and object extraction from text and visual data cannot deliver exciting information. In addition, the existing search engines support searching for things (objects or entities). It can interpret user queries expressed in natural languages, similar to semantic search engines (Malhas & Elsayed, 2020; Ben-Sghaier, Bakari & Neji, 2019; Romeo et al., 2019; Zeid, Belal & El-Sonbaty, 2020; Faris et al., 2022; Mozannar et al., 2019; Lahbari, El Alaoui & Zidani, 2018; Al-Shenak, Nahar & Halawani, 2019).

**Table 4:** Details of the initial search.

Databases	Initial Search
IEEE Xplore Digital Library	367
ScienceDirect	214
Springer Library	26
ACM Digital Library	10
Total	617

Information Extraction (IE) techniques must be used for unstructured or semi-structured data to extract the necessary details (Samy, Hassanein & Shaalan, 2019; Breja & Jain, 2022; Alothman & Wahab Sait, 2022; Hao et al., 2022; Gemirter & Goularas, 2021; Longpre, Lu & Daiber, 2021). To effectively handle and analyze massive amounts of multidimensional, unstructured data, it is essential to understand the strengths and limitations of the currently available IE approaches of data preprocessing, extraction, and transformation. Improving the effectiveness and precision of these IE systems is crucial (Almiman, Osman & Torki, 2020). However, the complexity and dimensionality of real-time data cause difficulties for ML-based techniques.

Users of relational databases (RDB) expect their natural language queries to provide more precise and specific responses (Utomo, Suryana & Azmi, 2020). Requests made by users in natural language must be translated into formal database queries such as SQL to retrieve data from RDBs (Mutabazi et al., 2021). They can recycle the application’s backend services. NLP can be employed to decipher users’ phrases and generate database-accessing URLs for application service requests.

Text streams on the Internet, mobile phone conversations, and IoT devices produce massive text databases (Boudjellal et al., 2021). The most common method for analyzing texts is text categorization, even though ML and NLP have become the most powerful tools. Text categorization may use Multilevel (MLL) or Multi-Class (MC) techniques. The instances in MC can be categorized into one class, whereas in MLL numerous labels may be applied to the same instance (Ahmed, Ahmed & Anto, 2017).

Multi-label data preprocessing for extensive data analysis is essential for solving MLL challenges. When MLL is applied to real-world data, it can become fraught with high-dimensional label space, label dependence, ambiguity, drifting, and unbalanced labels (Mohammed, Nasser & Harb, 1993; Hammo et al., 2002; Brini et al., 2009b; Brini et al., 2009a; Bdour & Gharaibeh, 2013; Abdelnasser et al., 2014). Concerns may arise from translating text from one language to another. The difficulty of providing a reasonable translation of a foreign language lies not in translating individual words but in grasping the intended meaning of entire sentences (Trigui, Belguith & Rosso, 2010; Zheng, 2002; Kurdi, Alkhaider & Alfaifi, 2014; Azmi & Alshenaifi, 2017; Reddy & Madhavi, 2017; Albarghothi, Khater & Shaalan, 2017; Sadek & Meziane, 2016). Each medium calls for a unique vocabulary and set of linguistic abilities. Word choice becomes more complicated when considering the subject matter and the target recipient (Ismail & Homsi, 2018; Akour et al., 2011; Al-Khawaldeh, 2019; Bakari & Neji, 2022; Nabil et al., 2017; Fareed, Mousa & Elsisi, 2013).

Sometimes, a word or phrase in one language may not precisely correspond to its counterpart in another language. Idiomatic expressions provide illustrative examples or figures of speech to clarify a concept. Furthermore, it is impossible to determine the meaning of a sentence by its words (Shaheen & Ezzeldin, 2014; Ray & Shaalan, 2016; Bouziane et al., 2015; Ezzeldin & Shaheen, 2012; Bakari, Bellot & Neji, 2016a). Preprocessing, sentence splitting, tokenizing, tagging, stemming, and lemmatizating are the processes of NLP-based QAS (Dodiya & Jain, 2013). NLP models demand a powerful computer to process massive and varied datasets. Compared with statistical ML models, NLP models are cumbersome in size and memory requirements (Bakari, Bellot & Neji, 2016b). It is expensive to re-create all medium NLP models for fresh data sets. Table 5 outlines the features of the Arabic QAS.

**Table 5:** Review outcome.

**Authors and Year**	**Type of QAS**	**Features**	**Dataset**
Abdelhafez, Khateeb & Yahya (2022)	Content	Employed machine learning model LSTM for QAS development.	AOL corpus was used.
Abdelnasser et al. (2014)	Content	Extracted keywords from the user query and retrieved the relevant content from the Holy Quran.	Holy Quran verses and the interpretation books
Alothman & Wahab Sait (2022)	Content	Employed Naïve Bayes algorithm for developing the ontological framework.	Corpus of 77 Arabic documents
Abouenour, Bouzoubaa & Rosso (2012)	Content	Designing QAS using Query Expansion and Passage Retrieval.	Not Available
Ahmed, Babu & Anto (2017)	Semantic	Text Retrieval Conference (TREC) and MRR (Mean Reciprocal Rank) were used.	Research Papers
Zheng (2002)	Ranking	Employed ML technology to answer a user query.	TREC
Akour et al. (2011)	Rule	Introduced a rule-based QAS using ML technique.	Web documents
Al Chalabi (2015)	Content	Designing QAS with machine learning model.	UIUC, 500 records for training and 500 for testing.
Al Chalabi, Ray & Shaalan (2015)	Pattern	Classification of questions using machine learning.	The corpus of 6,000 questions.
Albarghothi, Khater & Shaalan (2017)	Knowledge	Constructed an ontology to represent the user query in the resource description framework (RDF).	Web documents
Al-Chalabi, Ray & Shaalan (2015)	Semantic	Adding semantically equivalent keywords for Answer generation.	Dataset of 150 questions and answers.
Almiman, Osman & Torki (2020)	Content	Introduced deep learning-based QAS for online Arabic forums.	Online forum content
Al-Shenak, Nahar & Halawani (2019)	Semantic	Used support vector machine and latent semantic index for the query classification.	A dataset of 10,000 documents
Alsubhi, Jamal & Alhothali (2022)	Content	Design QAS using deep learning model.	Arabic-SQuAD and ARCD
Arbaaeen & Shah (2021)	Semantic	Employed a semantic approach for developing an Arabic QAS	Web documents
Azmi & Alshenaifi (2017)	Factoid	Used the rhetorical structure theory for developing the Arabic QAS.	Corpus of Arabic documents
Bakari, Bellot & Neji (2016b)	Logic	Employed text entailment method to handle open–domain queries.	Web documents
Bakari & Neji (2022)	Logic	Designed QAS using the conceptual graph.	Web corpus of questions and texts
Bdour & Gharaibeh (2013)	Semantic	Presented Yes / No responses for user queries.	A corpus of 20 Arabic documents
Biltawi, Tedmori & Awajan (2021)	Knowledge	Analyzed QAS considering 26 systems.	Not Available
Brini et al. (2009b)	Factoid	Used a web platform to develop the QAS.	Linguistic development environment
Brini et al. (2009a)	Factoid	Accepts the factoid questions and presents a response to them.	Linguistic development environment
Fareed, Mousa & Elsisi (2013)	Factoid	Developed a factoid QAS using query expansion techniques.	Web corpus
Faris et al. (2022)	Content	Proposed a healthcare QAS for patient and physician interaction.	Web documents
Al-Khawaldeh (2019)	Logic	Employed the text entailment for ranking the responses.	Web corpus
Hammo et al. (2002)	Content	Answering the user query based on the newspaper content.	Al-Raya newspaper content
Hamza et al. (2021)	Content	Built a classification system for classifying Arabic documents.	Web documents
Kurdi, Alkhaider & Alfaifi (2014)	Ranking	Employed a question analyzer to extract keywords from the user queries.	Arabic corpus of 39,660 words.
Lahbari, El Alaoui & Zidani (2018)	Semantic	Employed a hybrid Arabic part of speech and WordNet for query expansion.	TREC and CLEF
Malhas & Elsayed (2020)	Content	Introduced a content based QAS for the Holy Quran	The Holy Quran
Mohammed, Nasser & Harb (1993)	Knowledge	Presenting response based on the user query.	Not available
Mozannar et al. (2019)	Factoid	Term frequencies and neural comprehension models are used for the development.	1,395 Arabic questions
Mutabazi et al. (2021)	Logic	Used the text entailment method with the support of the search engine.	Web documents
Nabil et al. (2017)	Semantic	Built a model using an Arabic morphological analyzer.	Web corpus
Ray & Shaalan (2016)	Factoid	Employed a discourse relationship for developing the QAS.	Web documents
Romeo et al. (2019)	Content	Constructed a QAS using the ML technique.	Online forum content
Abuleil & Evens (2004)	Rule	Extracting entities from QAS using graphs and rules	Dataset of 335 documents
Sheker et al. (2016)	Content	Considered TF-IDF for domain specific QAS	Book of Fatwas
Trigui, Belguith & Rosso (2010)	Pattern	Identified information about the organization using web documents.	2,000 snippets of Google search engine and Wikipedia Arabic version
Zeid, Belal & El-Sonbaty (2020)	Semantic	Built a graph ontology for QAS.	Web corpus

 Mohammed, Nasser & Harb (1993) is one of the earliest attempts to develop a knowledge-based Arabic QAS. The bag of words method generated the outcome for a given query. However, the performance is based on the user query. Akour et al. (2011) developed a QAS, QARAB, to provide a short answer for a user query. They utilized the Al-Raya newspaper as a primary source for the QAS. In addition, a tagger is employed to extract nouns from the user query. Brini et al. (2009b) developed a factoid-based QAS in which they used a linguistic development environment for the QAS implementation. However, there is no exclusive experimentation outcome of the QAS. In addition, Brini et al. (2009a) proposed a QAS, QASAL, which accepts MSA as input and responds to the factoid questions.

In 2013, Bdour & Gharaibeh (2013) presented a formal model that responds with yes/no for the user query. They experimented with the model with 20 Arabic documents. The model was found to provide an optimal response. Another study by Abdelnasser et al. (2014) proposed a factoid-based QAS and evaluated the performance using TREC and CLEF datasets. The system extracts the relevant Quran verses according to the user query. The authors argued that the system had achieved 85% accuracy for the top three results.

Furthermore, Bakari, Bellot & Neji (2016b) proposed a logic-based approach to QAS development, employing the text entailment method. The authors stated that the model could analyze open-domain questions. Moreover, they employed the text entailment method. Bouziane et al. (2015) presented a neural Arabic QAS based on factoid questions (Malhas & Elsayed, 2020). The system was based on the term frequencies and the neural comprehension model. A dataset of 1,395 questions was employed for the experimentation. Another study by Zeid, Belal & El-Sonbaty (2020) provided a model for identifying information about the organization using web resources. The model achieved 90% accuracy for the top five answers. Lahbari, El Alaoui & Zidani (2018) developed a hybrid Arabic part of the speech model to transform the query to retrieve responses. They employed text retrieval conference (TREC) and cross-lingual evaluation form (CLEF) datasets for experimentation. Ahmed, Ahmed & Anto (2017) proposed a web-based QAS and ranked the documents using the term frequencies. Al-Shenak, Nahar & Halawani (2019) developed a web-based QAS named JAWEB in which they employed components like a user interface, question analyzer, passage retrieval, and answer extractor. The question analyzer parses each question and extracts keywords to answer the user queries. They employed an Arabic corpus containing 39,660 words. The results revealed that the model performed better than other Arabic QASs.

Samy, Hassanein & Shaalan (2019) developed a factoid QAS, LEMAZA, using the rhetorical structure theory. They applied the data preprocessing technique to extract the keywords from the Arabic queries. The model responded to the user queries based on the keywords. Breja & Jain (2022) recently developed an ontological framework to answer user queries. They compiled a dataset of 77 Arabic documents and transformed it into a word format. The documents were ranked based on user queries, similar to a search engine. Hao et al. (2022) developed an ML-based QAS to provide paragraph-level answers for user queries. They employed SVM and latent semantic index to classify user queries. Albarghothi, Khater & Shaalan (2017) developed an ontology and built the SPARQL queries to extract the answer from the RDF. The authors used the patterns of RDF to represent the user query. Longpre, Lu & Daiber (2021) developed a discourse-based approach to establish a QAS. A text parser deals with “why” and “how to” questions. In addition, they employed a set of heuristics to reduce the computational cost.

Furthermore, Ismail & Homsi (2018) compiled a dataset, DAWQAS, for training and testing the ML-based QAS. Utomo, Suryana & Azmi (2020) introduced a rule-based ML technique for answering Arabic questions. They employed the graph theory to rank the documents. Mutabazi et al. (2021) developed an answer extraction technique based on the text entailment method. The search engine is utilized to index the web pages. The ranked pages are re-ranked using the model. Bakari, Bellot & Neji (2016b) designed a logic-based QAS. They transformed the Arabic content into a conceptual graph to make a concept and relation.

Using an Arabic Morphological Analyzer, Nabil et al. (2017) built an Arabic QAS. In addition, an explicit semantic approach was employed for ranking the pages. They used query expansion and Khoja stemmer to extract keywords. Malhas & Elsayed (2020) introduced a QAS for the Holy Quran. It contained 207 questions and 1,762 answers.

 Mutabazi et al. (2021) presented an NLP-based QAS. They employed the text entailment method. Bdour & Gharaibeh (2013) designed a community QAS using the ML technique. They used a tree kernel and text representation method for parsing the content. Abdelnasser et al. (2014) constructed a semantic ontology for QAS. They employed graph theory to build the relationships between texts. Almiman, Osman & Torki (2020) introduced deep learning-based QAS using the term frequencies and similarity features.

The researchers identified the features of the existing QAS. However, most research works are based on closed domains and do not apply to other domains. The scarcity of the datasets is one of the reasons for the limitations in QAS development. In addition, there is a lack of unsupervised learning-based QAS which can be used in the open domain. The studies (Mohammed, Nasser & Harb, 1993; Hammo et al., 2002; Brini et al., 2009b; Brini et al., 2009a; Bdour & Gharaibeh, 2013; Abdelnasser et al., 2014; Trigui, Belguith & Rosso, 2010; Zheng, 2002; Kurdi, Alkhaider & Alfaifi, 2014; Azmi & Alshenaifi, 2017; Reddy & Madhavi, 2017; Albarghothi, Khater & Shaalan, 2017; Sadek & Meziane, 2016; Ismail & Homsi, 2018; Akour et al., 2011) reported similar results on QAS. Furthermore, the studies (Al-Khawaldeh, 2019; Bakari & Neji, 2022; Nabil et al., 2017; Fareed, Mousa & Elsisi, 2013; Shaheen & Ezzeldin, 2014; Ray & Shaalan, 2016; Bouziane et al., 2015; Ezzeldin & Shaheen, 2012; Bakari, Bellot & Neji, 2016a; Dodiya & Jain, 2013; Bakari, Bellot & Neji, 2016b) called for an effective Arabic QAS for accurate results.

## Conclusion

This study aimed to identify the challenges and limitations of Arabic QAS development. The researchers conducted a systematic literature review based on the PRISMA guidelines. Twenty-seven studies were selected using portals such as IEEE Explore, ACM, etc. Most research studies developed factoid-based QAS for the specific domain. The review results stress the importance of the unsupervised learning-based QAS to serve users with optimal responses. The complexity of the Arabic language creates challenges in QAS development. The researchers focued on implementing a framework that can specify symbols, relations, text, voice, and context, so a computer algorithm can apply language interpretation and produce meaningful conversations. The Transformer architecture is the core workhorse of NLP models, with the model’s scalability increasing quadratically with sequence length. One of the most notable problems in NLP is answering open-domain questions, which requires retrieving documents relevant to a particular query and utilizing them to construct an elaborate paragraph-length response. While significant progress has been made in factoid open-domain QA, where a single word, phrase, or object may resolve a query, long-form QAS has received significantly less attention. Therefore, QAS for unstructured and structured data requires extensive research studies. The natural language-based QAS can be employed in a wide range of applications. Using natural language processing in customer service leads to faster, more accurate customer replies. Researchers can use the study’s result to develop an Arabic QAS using the recent ML techniques.

For further research, the number of research articles to perform the critical analysis should be increased to obtain more valid and generalizable results.

## Additional Information and Declarations

### Data Availability

The following information was supplied regarding data availability: This is a systematic literature review article which is based on the PRISMA guidelines.
